# Effects of Microporosity and Surface Chemistry on Separation Performances of N-Containing Pitch-Based Activated Carbons for CO_2_/N_2_ Binary Mixture

**DOI:** 10.1038/srep23224

**Published:** 2016-03-18

**Authors:** Min-Sang Lee, Mira Park, Hak Yong Kim, Soo-Jin Park

**Affiliations:** 1 Department of Chemistry, Inha University, 100 Inharo, Incheon 402-751, Korea; 2 Department of Organic Materials and Fiber Engineering, Chonbuk National University, Jeonju 561-756, Korea; 3 Department of BIN Convergence Technology, Chonbuk National University, Jeonju 561-756, Korea

## Abstract

In this study, N-containing pitch-based activated carbons (NPCs) were prepared using petroleum pitch with a low softening point and melamine with a high nitrogen content. The major advantage of the preparation method is that it enables variations in chemical structures and textural properties by steam activation at high temperatures. The adequate micropore structures, appropriate chemical modifications, and high adsorption enthalpies of NPCs are favorable for CO_2_ adsorption onto carbon surfaces. Furthermore, the structure generates a considerable gas/N-containing carbon interfacial area, and provides selective access to CO_2_ molecules over N_2_ molecules by offering an increased number of active sites on the carbon surfaces. The highest CO_2_/N_2_ selectivity, i.e., 47.5, and CO_2_ adsorption capacity for a CO_2_/N_2_ (0.15:0.85) binary gas mixture, i.e., 5.30 wt%, were attained at 298 K. The NPCs also gave reversible and durable CO_2_-capturing performances. All the results suggest that NPCs are promising CO_2_ sorbents, which can meet the challenges of current CO_2_ capture and separation techniques.

Public concerns over the global warming caused by greenhouse gas (GHG) emissions have led to increased attention to cost- and energy-efficient techniques for CO_2_ capture and sequestration. Fossil-fueled power plants are considered to be responsible for about 40% of total carbon emissions, therefore CO_2_ capture from fossil-fueled power plants is a key objective in the ongoing effort to reduce the effects of GHGs on global climate change^1^,^2^,^3^,^4^,^5^,^6^,^7^. Three CO_2_ capture systems are currently used in conventional power plants: post-combustion, pre-combustion, and oxy-fuel capture systems. Post-combustion systems can be used for existing power plants, and can be directly applied to GHG emission reduction^8^,^9^,^10^,^11^. The emissions from current fossil-fueled power plants are multi-component gas mixtures, which consist mainly of N_2_. The CO_2_ concentration in the mixed gases in current post-combustion systems ranges from 10% to 15% by volume^12^,^13^,^14^,^15^,^16^.

A number of techniques have been studied for use in post-combustion capture systems, such as adsorption, physical and chemical absorption, cryogenics, and membrane methods. Recently, a liquid-phase amine scrubbing technique, which can achieve high-efficiency CO_2_ sequestration, has been used commercially, although it has the disadvantages of equipment corrosion, inherently high regeneration costs, and degradation in the gas mixture. Many researchers have focused on developing renewable solid sorbents that are competitive, energy efficient, and can be regenerated. However, for post-combustion systems in particular, no ideal sorbent is yet available, because of limitations such as low CO_2_ capture capacities for CO_2_/N_2_ binary mixtures, high costs, slow kinetics, low CO_2_/N_2_ selectivities, weak moisture resistance, and self-degradation properties^17^,^18^.

There are various types of CO_2_ sorbent, including zeolites, activated carbons (ACs), mesoporous silicas, and metal-organic frameworks (MOFs)^19^,^20^,^21^. Zeolites with low Si/Al ratios have high CO_2_/N_2_ selectivities, because of their high isosteric heat of adsorption for CO_2_ and their large internal electrical field gradients. MOFs have been studied as potential CO_2_ sorbents, because of their extremely high surface areas and typically high CO_2_ adsorption capacities. However, potential disadvantages of using zeolites and MOFs as CO_2_ sorbents include their limited physicochemical stabilities and sensitivities in the presence of water^22^. Recently, ACs have attracted interest as CO_2_ sorbents, because they have high mechanical hardness, thermal stability, and water resistance in conventional CO_2_ capture systems^23^,^24^,^25^,^26^,^27^,^28^. However, ACs are not ideal sorbents, because they have low CO_2_ adsorption capacities and CO_2_/N_2_ selectivities.

As in the case of ACs in general, pitch-based ACs with high-density graphitizable structures have diverse applications, including adsorption of gas- and liquid-phase pollutants, catalysts, and gas storage^29^,^30^,^31^. They are a complex blend of polyaromatic molecules and heterocyclic compounds, and can generally be obtained as by-products of petroleum industries. The use of pitch therefore has significant potential from the economic perspective.

CO_2_ and N_2_ are non-polar molecules with similar kinetic diameters, i.e., 3.30 and 3.64 Å, respectively. However, they have different quadrupole moments (2.85:1) and polarizabilities (1.5:1), and this enables the design of CO_2_-philic sorbents^32^. CO_2_ has an electron-deficient carbon atom; therefore, it is expected to have favorable interactions with electronegative species, leading to strong acid–base interactions, whereas N_2_ is not affected. This simple theory has led to the development of a plethora of nanoporous sorbents that incorporate nitrogen-rich chemical structures such as tetrazoles, triazines, imidazoles, phosphazenes, amines, and imides^33^.

In all these and many other situations, CO_2_-philicity is the main driving force for the selective capture of CO_2_ over N_2_, without taking into account any favorable interactions with CO_2_ that would selectively repel N_2_ molecules. The preparation of pitch-based carbon materials with tailored porous textures and surface chemistry is, from this perspective, essential for achieving optimum CO_2_ adsorption. The optimum pore size and a basic N-containing chemical structure are the key factors in obtaining ACs with the highest adsorption capacities and selectivities. N-containing ACs can be synthesized from solid amines with high initial nitrogen contents as the precursor, heat treatment of mixtures of ACs and nitrogen compounds, and chemical modification of carbon structures by nitrogen compounds at high temperatures^34^.

In this work, we developed a useful method for synthesizing N-containing pitch-based ACs (NPCs). It involves mixing pitch with a compound containing a large amount of nitrogen, followed by carbonization and steam activation. This method enables transformation of a mixture of raw pitch and melamine with a high nitrogen content into NPCs. The CO_2_ adsorption behaviors based on the chemical transformations and textural properties of the NPCs are discussed.

## Results and Discussion

An X-ray photoelectron spectroscopy (XPS) survey scan showed the presence of carbon, nitrogen, and oxygen in the samples, as shown in Figure S1. The slightly reduced intensity of the C1s peak for NPC-800 suggests that the carbon-containing groups, which were initially dominant in the raw pitch, were substituted by nitrogen-containing groups, or decomposed during activation of the pitch and melamine. The NPC-800 structure was further investigated by deconvolution of the C1s, O1s, and N1s peaks. The chemical structures of the raw pitch were altered by mixing with raw melamine and through the subsequent high-temperature activation process.

Figure S2 shows that the XPS C1s spectrum of melamine is completely different from those of the other samples. This spectrum has a small peak at 288.7 eV, a sharp peak at 287.4 eV, and a third one, of lower intensity, at 284.4 eV. This distribution agrees with the structure of melamine, in which two types of carbon atoms can be distinguished, the carbon atoms in the –C=N– species [binding energy (BE) around 287 eV] being the most abundant. The peak at 288.7 eV, i.e., with the highest BE among the three peaks, is assigned to the carbon attached to an oxygen atom in C=O. These carbonyl groups are assumed to be formed by surface oxidation, which could occur during the manufacture of pristine melamine^35^. It is evident from the XPS C1s peak of raw pitch that the raw pitch sample contains large quantities of C=C and C–C bonds and trace amounts of oxygen functional groups^36^. A comparison of the spectra of raw pitch and NPC-800 clearly shows that activation at high temperatures results in considerable changes in the C1s spectrum. High-temperature activation results in broadening of the C1s peak, because of the large number and variety of oxygen functional groups formed^37^. Furthermore, the higher-BE peaks mainly arise from species with carbon atoms bound to oxygen or nitrogen atoms. The carbon atoms in C–O or C=N groups have a BE of 286.6 eV, those in C=O or C–N species have a BE of 287.8 eV, and those in carboxyl groups have a BE of 289.2 eV^38^. The formation of various types of nitrogen–carbon structures could play a key role in causing stronger interactions between CO_2_ molecules and NPCs than between CO_2_ and raw pitch, which has no nitrogen species in its chemical structure. Nitrogen components in a graphitic carbon structure can act as basic sites, therefore access of CO_2_ molecules to the carbon surfaces is favored over access of N_2_ molecules.

The NPCs were prepared by heat treatment with steam in an air flow at temperatures of 600 °C and above. During this treatment, important chemical changes take place in the pitch, which is transformed into an infusible material. At low temperatures, i.e., around 300 °C, the consumption of aliphatic hydrogen atoms occurs, whereas at higher temperatures, aromatic hydrogens react to form oxygen-containing groups. Oxygen-containing groups that decompose between 300 and 600 °C give rise to new bonds between pitch molecules; when the temperature is higher than 600 °C, typical reactions such as cyclizations and condensation of carbon rings to form graphene-like structures take place^39^.

**Figure 1:**
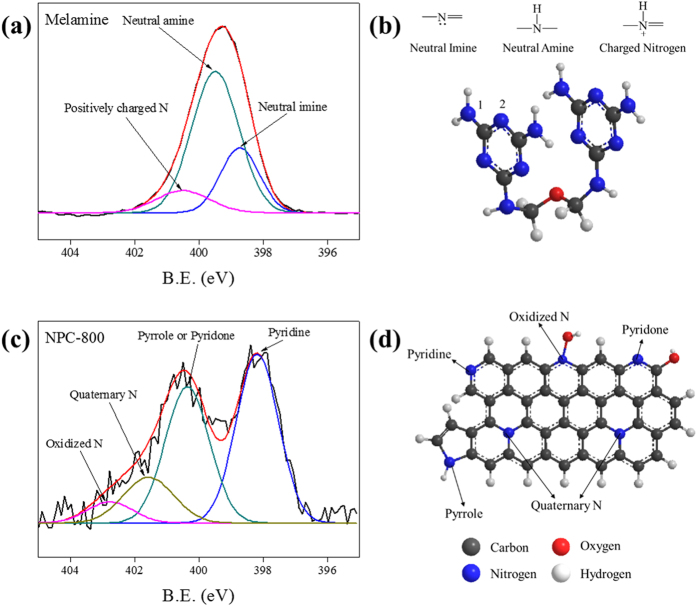
N1s XPS of pristine melamine and NPC-800 (**a,c**) and their structural representation showing N-containing functionalities on carbon surface based on N1s electrons (**b,d**).

The XPS O1s core-level spectrum of melamine (Figure S3) shows a well-defined peak at around 532 eV corresponding to ether bond formation by oxygen atoms, i.e., C–O–C species; this is in agreement with the chemical structure shown in Fig. 1b. A peak appears at 532 eV in the spectrum of raw pitch, which is attributed to oxygen atoms forming C–O single bonds^40^. The NPC-800 sample has a slightly wider O1s peak, indicating the presence of several types of surface oxygen functional groups, with BEs for O1s electrons ranging from 529 to 535 eV^41^.

Figure 1 shows the XPS N1s core-level spectra of raw melamine and NPC-800. Fitting of the melamine peak shows three contributions at BEs of 398.7, 399.4, and 400.5 eV. The chemical structure of melamine (Fig. 1b) and XPS studies performed with similar compounds such as polyaniline^42^ suggest that the three peaks can be assigned as shown in Fig. 1a. The peak at 398.7 eV can be attributed to neutral imine nitrogen atoms (^2^N in Fig. 1b), and the peak at 399.4 eV corresponds to neutral amine nitrogen atoms (^1^N in Fig. 1b). The peak at the highest BE (400.5 eV) can only be assigned to positively charged nitrogen species, as melamine does not contain additional nitrogenated species such as pyrroles or pyridines, which would also produce an XPS signal at this BE^43^. Figure S1 shows that the N1s XPS signal for raw pitch is almost negligible, because of the low nitrogen content. The N1s peak of NPC-800 cannot be interpreted using the same three species as those in melamine. Four peaks, at BEs of 398.1, 400.3, 401.5, and 402.8 eV, fit the N1s curve (Fig. 1c). It should be noted that the peak at the amine BE (i.e., 399.5 eV) does not appear. The peaks at BEs of 398.1 and 400.3 eV correspond to pyridine nitrogen functional groups (which are more stable than imines), and pyrrole or pyridone nitrogen functional groups, respectively. The species with a BE of 402.8 eV can be assigned to oxidized forms of nitrogen (see Fig. 1d), and the band at 401.5 eV corresponds to quaternary nitrogen^44^. The quaternary nitrogen species are located within the graphene layers that develop from condensation reactions that occur during carbonization. Similarly, when pitch is carbonized at a high temperature with melamine, chemical reactions occur between nitrogen species and the pitch molecules, resulting in the formation of cyclic nitrogen-containing structures, such as pyridines or pyrroles; these are the main nitrogen species detected when the material is carbonized, because condensation reactions take place. The chemical compositions of materials formed by activation of petroleum pitch and melamine at various temperatures are shown in Table S1. The data show that the nitrogen content decreased, whereas the carbon content increased, with increasing activation temperature from 600 to 900 °C, indicating selective removal of nitrogen during the thermal treatment. It can therefore be stated that the selective removal of nitrogen is highly dependent on the activation temperature. In addition, the graphene layered structure containing nitrogen atoms at inner positions has the excellent thermal stability of carbonaceous materials^45^. The thermogravimetric analysis (TGA) results in Figure S4 show that the NPCs are highly stable in the temperature range 30–900 °C in a N_2_ flow, unlike raw melamine, which undergoes major thermal decomposition at 250–370 °C. NPC-800 maintained 93% of its original weight even after heating to 900 °C. This high value is ascribed to its synthetic process, which involves steam activation at high temperature in an air flow^46^.

**Figure 2:**
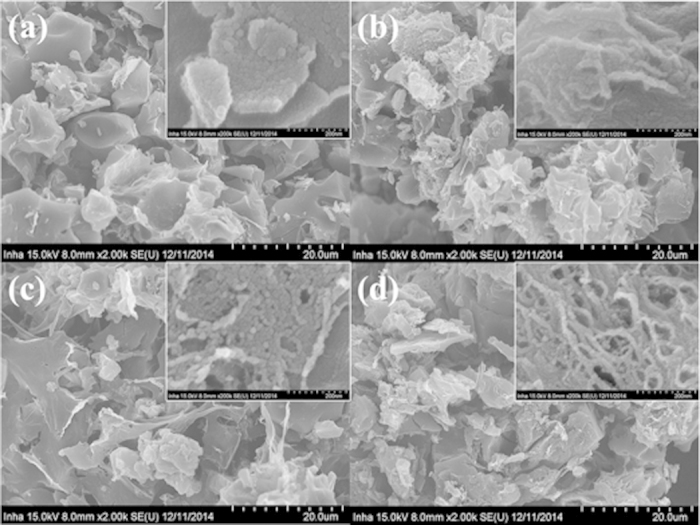
HR-SEM images of NPC-600 (**a**), NPC-700 (**b**), NPC-800 (**c**), and NPC-900.

The high-resolution scanning electron microscopy (HR-SEM) images in Fig. 2 show an irregular layered structure, which consists of pitch carbon chemically modified with melamine, and rough surfaces generated by steam activation. The high-resolution images show that the NPC samples have interconnected structures formed by chemically modified particles. Vacancies are formed as the activation temperature increases. These can lead to better diffusion of adsorbates and subsequently to favorable interactions with CO_2_.

**Figure 3:**
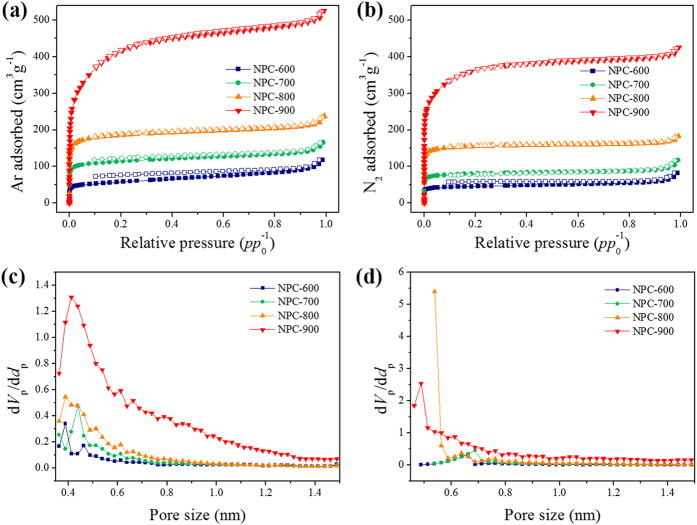
Ar and N_2_ adsorption–desorption isotherms (**a**,**b**) and Horváth–Kawazoe pore size distributions of NPCs calculated from Ar isotherms (**c**) and N_2_ isotherms (**d**).

The Ar adsorption isotherms at 87 K of the synthesized NPCs display typical type-I reversible adsorption profiles, indicating the presence of micropores on the carbon surfaces (Fig. 3a). Ar uptakes at 87 K by the NPC sorbents depend significantly on the sample activation temperature, implying that the textures of the carbon materials can be well controlled by changing the activation temperature. The porosity parameters are summarized in Table 1. The specific surface areas were 187, 384, 624, and 1344 m^2^ g^−1^ for NPC-600, NPC-700, NPC-800, and NPC-900, respectively; these values were calculated using an alternative linear form of the Brunauer–Emmett–Teller (BET) equation (Figures S5 and S6)^47^,^48^. The BET plots for the two series of NPCs using the two equations, i.e., the alternative and standard equations, are shown in Figures S5–S8. The two sets of plots obtained using the two equations differ considerably. The plots obtained using the alternative linear form show greater deviations from linearity and show asymptotic behavior at both low and high relative pressures, whereas the plots obtained over the same relative pressure range using the standard BET form are almost linear. The relative pressure range over which the BET equation gives linear plots can therefore be determined more precisely using the alternative approach instead of the standard one. It can also be observed that the constants *C* calculated for the NPCs using the standard BET equation are overestimated. The results in Table 1 imply that the specific surface area increases by a factor of two as the activation temperature is increased to 100 °C. The total pore volumes determined from Ar adsorption at a relative pressure *p*/*p*_0_ = 0.99 for NPC-600, NPC-700, NPC-800, and NPC-900 were 0.15, 0.21, 0.31, and 0.67 cm^3^ g^−1^, respectively; the micropore volumes calculated using the Dubinin–Radushkevich (DR) equation were 0.07, 0.14, 0.24, and 0.47 cm^3^ g^−1^, respectively. These results show that the samples prepared at higher activation temperatures have more abundant micropores, indicating that pore structure creation increases with increasing steam activation temperature. The maximum micropore sizes, obtained using the Horváth–Kawazoe (HK) method, are centered at 0.39, 0.44, 0.39, and 0.41nm for NPC-600, NPC-700, NPC-800, and NPC-900, respectively. The micropore sizes obtained from N_2_ isotherms using the HK method for NPC-600, NPC-700, NPC-800, and NPC-900 are 0.66, 0.69, 0.54, and 0.49 nm, respectively (Fig. 3c,d, Table 1). The calculated specific surface areas obtained from the N_2_ adsorption–desorption isotherms of the NPCs at 77 K are commensurate with those obtained based on Ar adsorption measurements (Fig. 3b and Table 1). NPC-900 shows broad peaks in the HK pore size distribution, suggesting an abundant and intricate microporous system. Pores larger than ~0.6 nm are only observed for NPC-900, implying that the greatest activation was achieved at the highest activation temperature (900 °C). We have also obtained HK pore size distribution using CO_2_ adsorption isotherms but it is not represented. CO_2_ is one of the standard molecules, along with N_2_ and Ar, used to analyze the pore size distributions of carbon materials, but it gives unreliable results in the presence of basic surfaces that can interact *via* specific interactions. Because the surfaces of NPCs contain large amounts of basic nitrogen components in the carbon structure, the HK method is unsuitable for use with CO_2_^49^.

**Figure 4:**
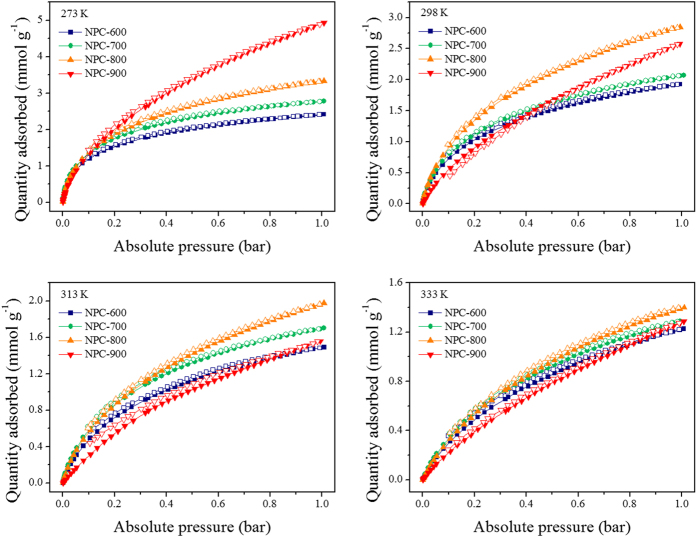
CO_2_ adsorption–desorption isotherms of NPCs at various temperatures.

CO_2_ adsorption–desorption isotherms for the NPC sorbents were also obtained at 273, 298, 313, and 333 K up to 1 bar. Figure 4 shows that the CO_2_ adsorption isotherms of the NPCs are type-I, according to the IUPAC classification. They are almost reversible over the entire pressure and temperature range investigated, meaning that the CO_2_ adsorbed on the NPCs can be desorbed simply by reducing the pressure. The amounts of CO_2_ adsorbed at 298 K are 1.95, 2.07, 2.84, and 2.57 mmol g^−1^ for NPC-600, NPC-700, NPC-800, and NPC-900, respectively (Table 2). As expected, the adsorption capacity decreases as the temperature increases. Nevertheless, the adsorption capacities at 333 K are still 1.22, 1.28, 1.39, and 1.28 mmol g^−1^ for NPC-600, NPC-700, NPC-800, and NPC-900, respectively; this indicates stable CO_2_ capture behavior even at elevated temperatures. The CO_2_ adsorption capacity of NPC-800 is higher than those of the other samples. This enhanced adsorption capacity stems from the high specific surface area, micropore volume, and *C* value of NPC-800. The CO_2_ adsorption capacities of NPC-900 are lower than those of NPC-800 at 298, 313, and 333 K, in spite of its higher specific surface area and micropore volume, indicating that the constant *C* played the most important role in this case. Although *C* constant does not provide a quantitative estimate of the enthalpy of adsorption, it is an indication of the magnitude of the adsorbent–adsorbate interaction energy^50^. Ar is inert and has a quadrupole moment of almost zero, therefore the constants *C* also indicate the magnitude of the surface energy, and can therefore be used to estimate adsorption potentials for other gases, CO_2_ in this case^51^.

It has been reported that sorbents that consist of N-containing carbon surfaces and have an optimum pore size have the potential to adsorb increased amounts of CO_2_. In the synthesized NPCs, melamine provides nitrogen-containing groups on the carbon surfaces, and these have a good affinity for CO_2_ molecules. In addition to the surface composition, the pore structures derived from the high-temperature activation process, e.g., micropores with optimum pore sizes and other sites, can affect the CO_2_ adsorption potential. Figure 4 shows that the isotherms bend slightly toward the vertical axis at pressures lower than 0.5 bar, whereas the isotherms become approximately linear at pressures greater than 0.5 bar. The subtle inflections on the isotherms suggest the presence of multiple adsorption sites for CO_2_ on NPCs. For the sake of simplicity, the adsorption sites are classified into two types: active adsorption sites in micropores with the optimum pore size (site 1), and other sites (site 2)^52^.

Based on the above considerations, the dual-site Langmuir (DSL) model was used to describe the adsorption equilibria of CO_2_ on the NPCs. Nonlinear parameter estimation was performed using a combined fitting method. The measured isotherm data for the NPCs can be appropriately correlated with the DSL model (Figure S9); the corresponding estimated values of the adsorption parameters are collected in Table S2. The adsorption equilibrium constant for the first site (*b*_1_) is significantly larger than that for the second site (*b*_2_) at all temperatures, implying a much stronger affinity for CO_2_ at site 1 than site 2. The contributions of site 1 and site 2 to CO_2_ adsorption on NPCs, based on the fitting data, are shown in Figure S11. CO_2_ is predominantly adsorbed at site 1 at low pressures and achieves adsorption saturation at about 0.3 bar. In contrast, the amount adsorbed at site 2 continues to accumulate as the pressure increases. CO_2_ adsorption at site 1 can therefore be attributed to micropore filling with appropriate pore width, whereas that at site 2 mainly involves adsorption on pores other than micropores with appropriate pore width. The data in Table S2 show that NPC-900 has the highest *q*_*sat,A*_ value, indicating the highest saturated CO_2_ adsorption capability at site 1. The pore size distribution of NPC-900, above 0.6 nm, which is about twice as large as the kinetic diameter of CO_2_ (0.33 nm), is broader than those of the other NPCs. The large amount of micropores of size 0.6 nm results in strong adsorption of CO_2_ molecules and such micropores has been shown to be selective for CO_2_ adsorption^53^. However, the correlation between *q*_*sat,A*_ and the CO_2_ adsorption performances (e.g., adsorption capacity and selectivity) clearly shows that other factors have a greater effect on the CO_2_ adsorption performances than *q*_*sat,A*_ does. As discussed above, the differences among the surface energies, according to the activation temperatures, make the main contributions to the adsorption capacities and CO_2_/N_2_ selectivities.

**Figure 5:**
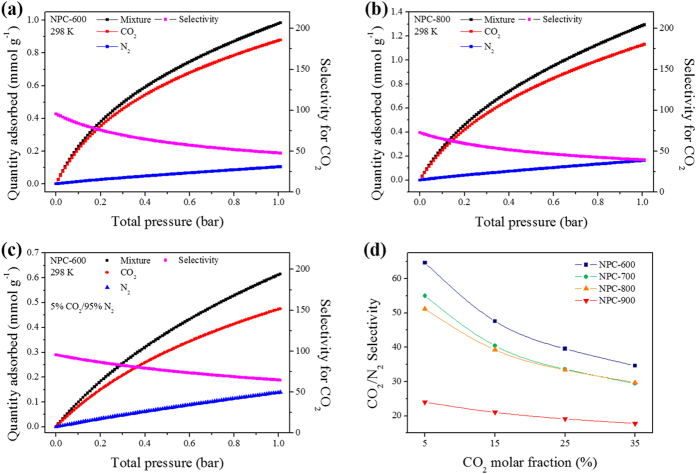
Binary IAST isotherms and selectivities for CO_2_ over N_2_ for NPC-600 (**a**) and NPC-800 (**b**) in CO_2_/N_2_ gas mixture (0.15:0.85), and results for NPC-600 in CO_2_/N_2_ gas mixture (5:95) (**c**). Ideal selectivity as function of CO_2_ molar fraction for NPCs at 298 K and total pressure of 1 bar (**d**).

For post-combustion CO_2_ adsorption, CO_2_ must be selectively adsorbed over N_2_. The ideal adsorbed solution theory (IAST), developed by Myers and Prausnitz, is frequently used to predict multicomponent isotherms on the basis of single-component isotherms^54^,^55^. On this basis, the selectivities were calculated for a 0.15:0.85 CO_2_/N_2_ mixture (Fig. 5a,b, Figures S12–S15, Table 2). The IAST CO_2_/N_2_ selectivities of the NPCs ranged from 34 to 107 at 273 K, from 21 to 47 at 298 K, from 23 to 53 at 313 K, and from 21 to 44 at 333 K. At all temperatures, the CO_2_/N_2_ selectivities of NPC-600 and NPC-700 were better than that of NPC-800, although it had the highest CO_2_ adsorption capacities based on the CO_2_ adsorption–desorption isotherms.

This unexpected behavior cannot be explained only by conventional CO_2_ affinities. Instead, a new concept, namely N_2_-phobicity, should be considered^56^. As discussed above, NPC-800 has the highest *C* value and abundant micropores for CO_2_ molecules, leading to a significant affinity for CO_2_ molecules. However, an activation temperature greater than 700 °C leads to a considerable decrease in the amount of nitrogen, which endows the carbon surfaces with N_2_-phobicity. The data in Table S1 show that the nitrogen contents of NPC-800 and NPC-900 are much smaller than those of NPC-600 and NPC-700 (Table S1). The higher N_2_-phobicities of NPC-600 and NPC-700 (compared with that of NPC-800) result in better CO_2_/N_2_ selectivities, for a CO_2_:N_2_ ratio of 0.15:0.85, despite their lower CO_2_ capacities based on CO_2_ adsorption isotherms (compared with NPC-800) (Fig. 5a,b). These results imply that N_2_-phobicity generated by N-containing surfaces can be a dominant factor in determining CO_2_/N_2_ selectivities. NPC-900 had the lowest *C* constant from the BET equation, and the lowest nitrogen content among all the prepared samples. This endows NPC-900 with a low CO_2_ affinity and N_2_-phobicity, resulting in the lowest CO_2_/N_2_ selectivities. The excellent CO_2_ adsorption capacity of NPC-800, based on its CO_2_ adsorption–desorption isotherm, suggests that it is potentially an excellent sorbent for CO_2_; however, NPC-600 and NPC-700 are the best sorbents for gas separation applications, especially for selective adsorption of CO_2_ over N_2_.

The ideal selectivity for CO_2_ over N_2_ at a pressure of 1 bar was plotted as a function of the CO_2_ molar fraction; the plots are shown in Fig. 5c,d and Figures S16–S19. For all the NPCs, the selectivities for CO_2_ decrease with increasing the CO_2_ molar fraction (Fig. 5d). The predicted selectivity for CO_2_ on NPC-600 ranges from 34.6 to 64.6, depending on the CO_2_ molar fraction in the CO_2_/N_2_ mixture. The other NPCs showed lower CO_2_ selectivities, although they have stronger adsorption potentials for CO_2_. This is because the NPCs, except NPC-600, have higher N_2_ adsorption capacities, and this decreases the CO_2_ selectivity (Figure S10). The higher nitrogen content of NPC-600 (compared with the other NPCs) contributes to its higher CO_2_ adsorption selectivity over N_2_. The IAST selectivity value of NPC-600 at 298 K or similar temperatures is significantly higher than, or at least comparable to, those of recently reported porous nanomaterials^57^,^58^,^59^,^60^,^61^,^62^. More detailed comparisons are shown in Table S3. The probable reason is that the interactions of the NPC samples with CO_2_ molecules are strong, because of the large amount of micropores with sizes that fit CO_2_ molecules, and the basic surface provided by the N-containing species derived from chemical restructuring of petroleum pitch with melamine.

**Figure 6:**
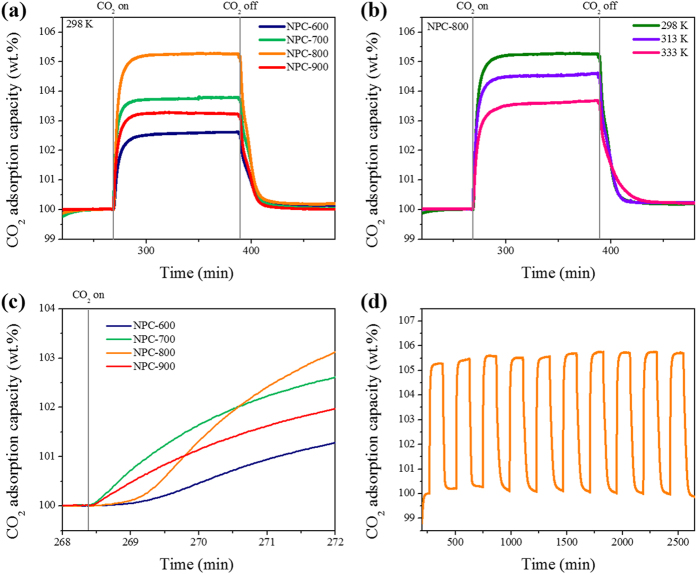
Time-dependent CO_2_ adsorption curves for CO_2_/N_2_ (0.15:0.85) gas mixture with NPCs at 298 K (**a**) and results for NPC-800 at various temperatures (298, 313, and 333 K) (**b**). Results for initial section for comparison of adsorption rates (**c**), and cycling of CO_2_ adsorption from gas mixture following regeneration by N_2_ purging (**d**).

CO_2_ adsorption experiments were conducted with a CO_2_/N_2_ (0.15:0.85) binary mixture, using TGA, to investigate potential applications of NPCs in post-combustion CO_2_ capture. Adsorption was achieved by exposing the sorbents to a stream of 15% (v/v) CO_2_ in N_2_ at 298, 313, and 333 K; these conditions approximately replicate conventional CO_2_ capture and sequestration systems. After adsorption, a pure N_2_ stream was used to regenerate the sorbents. Figure 6a shows that the NPCs reached CO_2_ adsorption equilibrium within 40 min at 298 K. CO_2_ capacities of 2.68, 3.84, 5.30, and 3.30 wt% were achieved using NPC-600, NPC-700, NPC-800, and NPC-900, respectively. The NPCs were regenerated in 40 min under a N_2_ flow. CO_2_ capacities of 2.01, 2.50, 4.65, and 2.01 wt% were obtained with NPC-600, NPC-700, NPC-800, and NPC-900, respectively, in 20 min at 313 K; the NPCs were completely regenerated in 20 min. The CO_2_ uptakes at 333 K for NPC-600, NPC-700, NPC-800, and NPC-900 were 1.23, 2.36, 3.73, and 1.67 wt%, respectively (Figure S20 and Table S4).

The results in Fig. 6b and Table S4 show that the CO_2_ adsorption capacities at all temperatures gradually decrease with increasing temperature, because of the thermodynamically unfavorable conditions at higher temperatures. Solid sorbents with high adsorption enthalpies have the advantages of high selectivities and capacities for CO_2_/N_2_ (0.15:0.85) binary mixtures, although material regeneration is a concern^63^. The recycling properties of the NPCs were examined by performing 10 CO_2_ adsorption–desorption cycles at 298 K for 120 min, followed by application of a pure N_2_ stream at the same temperature. At every cycle, the regenerated material retained its stability (Fig. 6d). We also measured the adsorption rates of the fully activated solids in a CO_2_/N_2_ (0.15:0.85) binary mixture with a flow rate of 60 mL min^−1^ at 298, 313, and 333 K (Fig. 6c, Table S4). NPC-800 showed the highest initial adsorption rates, i.e., 1.63, 1.66, and 0.98 wt% min^−1^ at 298, 313, and 333 K, respectively. The adsorption potential factor, *C* constant, calculated from the alternative BET equation, can provide an explanation for these adsorption rate results. The adsorption rate behaviors of the NPCs correlate well with the values of *C* calculated using the alternative BET equation.

## Conclusions

We developed highly effective AC sorbents based on a petroleum pitch support for selective CO_2_ capture over N_2_. The synthesis of N-containing ACs by steam activation provides controllable structures, with porous textures and chemical natures that can be precisely tailored. The modified chemical and textural structures enable preferential CO_2_ adsorption over N_2_, and generate considerable gas/N-containing carbon interfacial areas. The structure also provides surfaces with high CO_2_ adsorption potentials. When the sorbents were evaluated for CO_2_ capture applications, the as-prepared NPC-800 displayed outstanding adsorption features at all experimental temperatures. More specifically, the highest adsorption capacity obtained was 5.30 wt% for a CO_2_/N_2_ (0.15:0.85) binary mixture at 298 K. Additionally, the superior IAST selectivity achieved for CO_2_/N_2_ (0.15:0.85), namely 47.5 at 298 K, was also attained with NPC-600. All the results suggest that NPCs are highly sorptive, reversibly dynamic, and regenerable sorbents for selective CO_2_ capture at all experimental temperatures. These NPCs therefore have potential applications in post-combustion CO_2_ capture. The novel physical and chemical structures observed in this study may also represent a new type of sorbent for CO_2_, and should be the subject of further investigation.

## Methods

### Pre-treatment of petroleum pitch

Petroleum pitch (GS Caltex Co.) was pre-treated for use as a carbon precursor; it had a softening point of 423 K and did not contain any nitrogen. The petroleum pitch was ball-milled at 200 rpm for 5 h. The resulting powder was mixed with hydrochloric acid solution (30%, Sigma Aldrich Co.) and stirred continuously for 6 h to remove residual metal particles and other impurities. The powder was recovered and washed several times with ethanol and water. The pre-treated petroleum pitch was obtained by drying in a vacuum oven at 80 °C.

### Synthesis of NPCs

Pre-treated petroleum pitch and melamine (Sigma Aldrich Co.) were used as a supporting material and a nitrogen source, respectively. NPCs were synthesized by mixing pre-treated pitch with melamine in an organic solvent, followed by high-temperature steam activation. More specifically, pre-treated pitch (1.2 g) was dissolved in tetrahydrofuran (50 mL; Sigma Aldrich Co.), melamine (2.8 g) was added to the solution, and the mixture was continuously stirred at 300 rpm and 50 °C for 12 h. The mixture was dried in an oven at 80 °C until the solvent had completely evaporated, resulting in a dry black solid. The physical mixture was carbonized and activated by heating.

The dried solid was steam-activated as follows. (1) The sample was placed in a tube furnace with a syringe for the injection of steam molecules. (2) The furnace temperature was gradually increased to the desired temperature (600, 700, 800, and 900 °C) at a rate of 2 °C min^−1^. As it reached the desired temperature, steam molecules were injected from the syringe into the tube furnace at an injection rate of 6 mL h^−1^. Steam injection at the desired temperature was maintained for 1 h under an air flow, while maintaining a slightly increased pressure (about 1.2 bar), to increase the activation energy of the steam molecules. A series of NPCs were obtained by varying the activation temperature, i.e., 600, 700, 800, and 900 °C; they are denoted by NPC-600, NPC-700, NPC-800, and NPC-900, respectively.

### Characterization

The morphologies of the samples were examined using HR-SEM (SU8010, Hitachi Co., Ltd., Japan). The samples were attached to the observation platform and sprayed with platinum vapor under high vacuum for about 10 min. Elemental analysis of the samples was performed using an element analyzer (Thermo EA 1112, Thermo Scientific Co., USA) to determine their carbon, hydrogen, nitrogen, and oxygen contents (%). The chemical structures of the sorbents were investigated using XPS (K-Alpha, Thermo Scientific Co., USA) with a VG Scientific ESCALAB MK-II spectrometer equipped with a Mg Kα (1253.6 eV) X-ray source and a high-performance multichannel detector, which was operated at 200 W. TGA was performed using a thermogravimetric analyzer (TG209F3, Netzsch Co., Germany) by heating the samples to 900 °C at a rate of 10 °C min^−1^ under an inert N_2_ flow. The N_2_ adsorption–desorption isotherms at 77 K were measured to investigate the textural properties such as the specific surface area and pore structure, using an automated adsorption apparatus (BELSORP, BEL Co., Japan). Before the measurements, the samples were pre-treated in a vacuum at 200 °C for 12 h to release any remaining moisture or organic species. The pore structure was examined more precisely using Ar adsorption isotherms at 87 K, because the size and quadrupole moment of Ar are lower than those of nitrogen.

The adsorption–desorption isotherms were used to obtain the pore parameters, i.e., the specific surface area, pore volume, pore size, and magnitude of the adsorption enthalpy (based on *C* constant). The specific surface area was calculated using the BET equation, and total pore volumes were obtained from the amount of N_2_ or Ar adsorbed at a relative pressure (*p*/*p*_0_) of 0.99. The micropore volumes were calculated using the DR equation. The pore size distributions of the NPCs were calculated using the HK method. The CO_2_ and N_2_ adsorption–desorption isotherms of the NPCs at 273, 298, 313, and 333 K were also measured to investigate the CO_2_ adsorption behavior, using an automated adsorption apparatus (BELSORP, BEL Co., Japan).

### Cycling CO_2_ adsorption experiments for CO_2_/N_2_ (0.15:0.85) binary mixture

The fundamental kinetics of CO_2_ adsorption–desorption for a CO_2_/N_2_ (0.15:0.85) binary mixture using the NPC sorbents were investigated using TGA (Pyris 1, Perkin Elmer Co., Ltd., USA). In a typical test, the sample (10 mg) was placed in a ceramic balance and dried in an inert flow of N_2_ at 110 °C for 5 h before adsorption. The sample was cooled to 298, 313, or 333 K, and adsorption was performed in a stream of 15% CO_2_ in N_2_, which mimics post-combustion systems. The weight of the sorbent sample and the adsorption temperature were recorded continuously. In all experiments, the flow rates of the reactant gas (CO_2_) and inert gas (N_2_) were set at 60 mL min^−1^, and the stream of CO_2_ was kept constant for 60 min to fully adsorb CO_2_ on the sorbents. After adsorption, the sorbent was regenerated using a N_2_ flow. The recyclabilities of the sorbents were determined by repeating the adsorption–desorption process 10 times. The adsorption rates of the NPCs were calculated at the initial point of the adsorption kinetics.

## Additional Information

**How to cite this article**: Lee, M.-S. *et al.* Effects of Microporosity and Surface Chemistry on Separation Performances of N-Containing Pitch-Based Activated Carbons for CO_2_/N_2_ Binary Mixture. *Sci. Rep.* **6**, 23224; doi: 10.1038/srep23224 (2016).

## Supplementary Material

Supplementary Information

## Figures and Tables

**Table 1:** Textural and surface properties of samples studied.

Specimens		BET surface area (m^2^ g^−1^)	Total pore volume (cm^3^ g^−1^)	Micro pore volume (cm^3^ g^−1^)	Pore size (nm)	*C* constant
NPC-600	Ar	187	0.15	0.07	0.39	569
	N_2_	177	0.13	0.07	0.66	1324
NPC-700	Ar	384	0.21	0.14	0.44	1263
	N_2_	315	0.18	0.12	0.69	1525
NPC-800	Ar	624	0.31	0.24	0.39	1485
	N_2_	619	0.28	0.24	0.54	2127
NPC-900	Ar	1344	0.67	0.47	0.41	138
	N_2_	1256	0.65	0.46	0.49	269

The specific surface areas, total pore volumes, and *C* values were calculated from Ar and N_2_ adsorption isotherms using the alternative BET equation. The micropore volume and pore size were obtained using the D-R equation and H-K method.

**Table 2:** CO_2_ adsorption performances, i.e., adsorption capacities and IAST selectivities, of NPCs at various temperatures.

		273 K	298 K	313 K	333 K
NPC-600	CO_2_ adsorption capacity (mmol g^−1^)	2.41	1.95	1.49	1.22
	IAST selectivity	77.6	47.5	49.1	44.2
NPC-700	CO_2_ adsorption capacity (mmol g^−1^)	2.78	2.07	1.70	1.28
	IAST selectivity	107.5	40.3	53.1	40.0
NPC-800	CO_2_ adsorption capacity (mmol g^−1^)	3.34	2.84	1.97	1.39
	IAST selectivity	58.1	39.9	50.4	39.6
NPC-900	CO_2_ adsorption capacity (mmol g^−1^)	4.93	2.57	1.55	1.28
	IAST selectivity	34.7	21.8	23.3	21.6

CO_2_ adsorption capacities were measured from experimental CO_2_ isotherms at 1 bar. Selectivities were calculated for a simulated CO_2_/N_2_ mixture (0.15:0.85) using the IAST.
