# Supplementary material for: Effects of Microporosity and Surface Chemistry on Separation Performances of N-Containing Pitch-Based Activated Carbons for CO2/N2 Binary Mixture
Source: Sci Rep. 2016 Mar 18;6:23224. doi: 10.1038/srep23224 (PMC4796795; doi:10.1038/srep23224)
Supplement: Supplementary Information [file srep23224-s1.pdf]

Supporting Information

Effects of Microporosity and Surface Chemistry on Separation Performances of N-Containing Pitch-Based Activated Carbons for CO_2/N_2 Binary Mixture

Min-Sang Lee[1], Mira Park[2], Hak Yong Kim[3] & Soo-Jin Park[1,*]

[1]*Department of Chemistry, Inha University, 100 Inharo, Incheon 402-751, Korea.*

[2]*Department of Organic Materials and Fiber Engineering, Chonbuk National University, Jeonju 561-756, Korea.*

[3]*Department of BIN Convergence Technology, Chonbuk National University, Jeonju 561-756, Korea.*

*Corresponding author. Tel.: +82-32-876-7234; Fax: +82-32-876-7234.

E-mail address: sjpark@inha.ac.kr (S.-J. Park)

Plotting of BET equation using alternative approach

The standard method for calculating surface areas is based on the BET equation, although the theoretical model underlying the BET equation is quite crude. Use of the traditional linear form of the BET equation gives rise to considerable degrees of uncertainty and subjectivity in determining the relative pressure range over which the equation can be applied; this problem is exacerbated in the case of microporous carbons, for which the applicable relative pressure range is narrower. The BET equation can be expressed as

$$V = \frac{V_{sat}Cp}{(p_0 - p)[1 + (C - 1)(p/p_0)]}, \tag{1}$$

where V is the volume of gas adsorbed at a pressure p, V_{sat} is the volume of gas required to cover a monolayer on the surface of the sorbent, p_0 is the saturation vapor pressure of the adsorbate at the adsorption temperature, and C is a parameter that can be used to estimate the magnitude of the adsorption enthalpy, and is given by the following equation:

$$C = A\,exp\left(\frac{E_1 - E_L}{RT}\right). \tag{2}$$

Here, E_1 is the mean enthalpy of adsorption of the first layer and E_L is the enthalpy of liquefaction of the adsorbate. The linear form of the BET equation that is generally used is

$$\frac{X}{V(1-X)} = \frac{1}{V_{sat}C} + \left(\frac{C-1}{V_{sat}C}\right)X , \qquad (3)$$

where $X = p/p_0$. The intercept and slope of the linear plot can be used to determine V_{sat}, which is a measure of the surface area and the parameter C.

Use of the original BET equation [Eq. (3)] presents several difficulties, the most important being the uncertainty in the determination of the relative pressure range over which the equation can be applied. Brunauer, Emmett, and Teller recommended the use of a relative pressure range of 0.05–0.35. However, it has been pointed out that the most appropriate range for each particular material occurs in the region where the equation becomes linear (Sing *et al.*, 1985).

The BET equation [Eq. (3)] can be written in an alternative linear form (Keii et al., 1961) as follows:

$$\frac{1}{V(1-X)} = \frac{1}{V_{sat}} + \frac{1}{V_{sat}C}\left(\frac{1-X}{X}\right). \qquad (4)$$

This linear form of the BET equation was used in the present study to calculate the BET parameters, i.e., the specific surface area and the constant C, and to determine the relative pressure range over which the BET equation is applicable in the case of carbon-based materials (Parra Soto *et al.*, 1995).

Calculation of CO_2/N_2 adsorption selectivity

The ideal adsorbed solution theory (IAST) is frequently used to estimate the composition of an adsorbed phase from the pure-component isotherm data, and to predict the selectivity for binary mixtures of CO_2/N_2 (Myers and Prausnitz, 1965). Here, the experimental data for pure-component isotherms for CO_2 and N_2 at 273, 298, 313, and 333 K were fitted using single-site Langmuir (SSL) or dual-site Langmuir (DSL) models:

$$\text{SSL:} \quad q = \frac{q_{sat} b p}{1 + bp}, \tag{5}$$

$$\text{DSL:} \quad q = \frac{q_{sat,A} b_A p}{1 + b_A p} + \frac{q_{sat,B} b_B p}{1 + b_B p}, \tag{6}$$

where q is the adsorption capacity, q_{sat} is the saturated adsorption capacity, and b is the coefficient of the Langmuir equation. In the DSL model, the subscripts A and B indicate the parameters for adsorption sites A and B, respectively.

After calculating the molar fraction (x) of component a in the adsorbed phase using the IAST theory, the adsorption selectivity can be calculated; it is defined as

$$S = \frac{x_a / y_a}{x_b / y_b}, \tag{7}$$

where x_a and y_a are the molar fractions of component *a* in the adsorbed phase and bulk phase, respectively; x_b and y_b are the molar fractions of component *b* in the adsorbed phase and bulk phase, respectively.

References

S1. Sing, K. S. W. *et al.* Physical and biophysical chemistry division commission on colloid and surface chemistry including catalysis. *Pure Appl. Chem.* **57**, 603-619 (1985).

S2. Keii, T., Takagi, T. & Kanetaka, S. A new plotting of the BET method. *Anal. Chem.* **33**, 1965 (1961).

S3. Parra Soto, J. B., De Sousa, J. C., Bansal, R. C. & PisMartínez. J. J. Characterization of activated carbons by the BET equation: an alternative approach. *Adsorpt. Sci. Technol.* **12**, 51-66 (1995).

S4. Myers, A. L. & Prausnitz, J. M. Thermodynamics of mixed-gas adsorption. *AIChE J.* **11**, 121-127 (1965).

S5. Caldwell, S. J. *et al.* Carbon dioxide separation from nitrogen/hydrogen mixtures over activated carbon beads: adsorption isotherms and breakthrough studies. *Energ. Fuel.* **29**, 3796-3807 (2015).

S6. Zhou, X. *et al.* Enhanced separation performance of a novel composite material GrO@ MIL-101 for CO_2/CH_4 binary mixture. *Chem. Eng. J.* **266**, 339-344 (2015).

Table S1. Chemical compositions of NCPs prepared at different temperatures.

Specimens	Chemical composition (wt%)				N/C[a]
	N	C	H	Other	
NPC-600	10.52	76.19	1.78	11.51	0.138
NPC-700	9.25	76.13	1.38	13.24	0.122
NPC-800	5.28	80.23	0.85	13.64	0.066
NPC-900	3.14	87.97	0.56	8.33	0.036

[a] Weight ratios

Table S2. Estimated values of adsorption parameters for fitting of isotherm data for CO_2 on NPCs using dual-site Langmuir model.

	Temp.	$q_{sat,A}$ (mmol g^{-1})	b_A bar^{-1}	$q_{sat,B}$ (mmol g^{-1})	b_B bar^{-1}
NPC-600	273 K	0.94	52.42	2.21	2.24
	298 K	0.76	22.24	2.23	1.34
	313 K	0.57	11.31	1.92	0.98
	333 K	0.33	7.89	2.14	0.75
NPC-700	273 K	0.95	59.07	2.52	2.54
	298 K	0.70	23.61	2.33	1.45
	313 K	0.48	18.50	2.05	1.51
	333 K	0.44	7.79	2.08	0.75
NPC-800	273 K	0.98	44.90	3.68	1.75
	298 K	0.83	19.28	4.21	0.95
	313 K	0.52	14.36	3.18	0.86
	333 K	0.21	10.97	2.56	0.87
NPC-900	273 K	1.01	24.70	9.66	0.68
	298 K	0.76	7.30	10.07	0.23
	313 K	1.32	2.28	21.91	0.02
	333 K	1.54	0.69	1.54	0.69

Table S3. Comparison of experimental CO_2 adsorption capacities and CO_2/N_2 selectivities calculated using the IAST method for CO_2/N_2 binary gas mixture (0.15:0.85) reported in the literature and found in present work.

	CO_2 adsorption capacity at 1bar (mmol g^{-1})			CO_2/N_2 selectivity				Ref.
	293 K	296 K	298 K	CO_2/N_2 fraction	293 K	296 K	298 K	
UiO-66-($COONa)_2$-Ex	-	-	1.98	0.15:0.85	-	-	33.2	57
OAC-1	-	-	3.46	0.5:0.5	-	-	26.5	58
Modified activated carbon	-	-	-	0.1:0.9	-	-	26.8	59
PIN1	-	-	1.22	0.15:0.85	-	-	31.0	60
ZJNU-45a	-	4.77	-	0.15:0.85	-	16.4	-	61
$[Cu(tba)_2]_n$	1.95	-	-	0.15:0.85	45	-	-	62
NPC-600	-	-	1.95	0.15:0.85	-	-	47.5	This work
NPC-700	-	-	2.07	0.15:0.85	-	-	40.3	This work
NPC-800	-	-	2.84	0.15:0.85	-	-	39.9	This work
NPC-900	-	-	2.57	0.15:0.85	-	-	21.8	This work

Comparison was performed based on recently published results for CO_2 performances at 298 K or similar temperatures.

Table S4. CO_2 adsorption performances of NPCs for CO_2/N_2 binary gas mixture (0.15:0.85) determined using TGA method.

		298 K	313 K	333 K
NPC-600	CO_2 adsorption capacity (wt.%)	2.68	2.01	1.23
	Adsorption rate (wt.% min^{-1})	0.65	0.86	0.60
NPC-700	CO_2 adsorption capacity (wt.%)	3.84	2.50	2.36
	Adsorption rate (wt.% min^{-1})	1.44	1.06	1.34
NPC-800	CO_2 adsorption capacity (wt.%)	5.30	4.65	3.73
	Adsorption rate (wt.% min^{-1})	1.63	1.66	0.98
NPC-900	CO_2 adsorption capacity (wt.%)	3.30	2.01	1.67
	Adsorption rate (wt.% min^{-1})	1.03	1.39	0.88

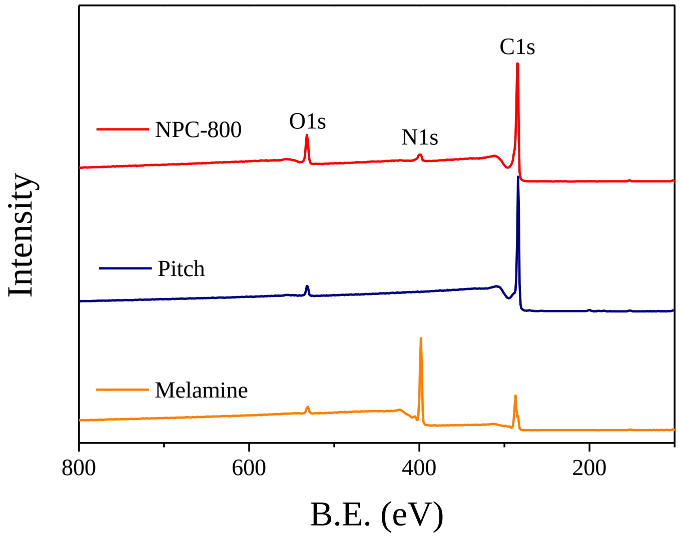

Figure S1. Survey scan XPS spectra for precursors (pristine melamine and pitch) and NPC-800.

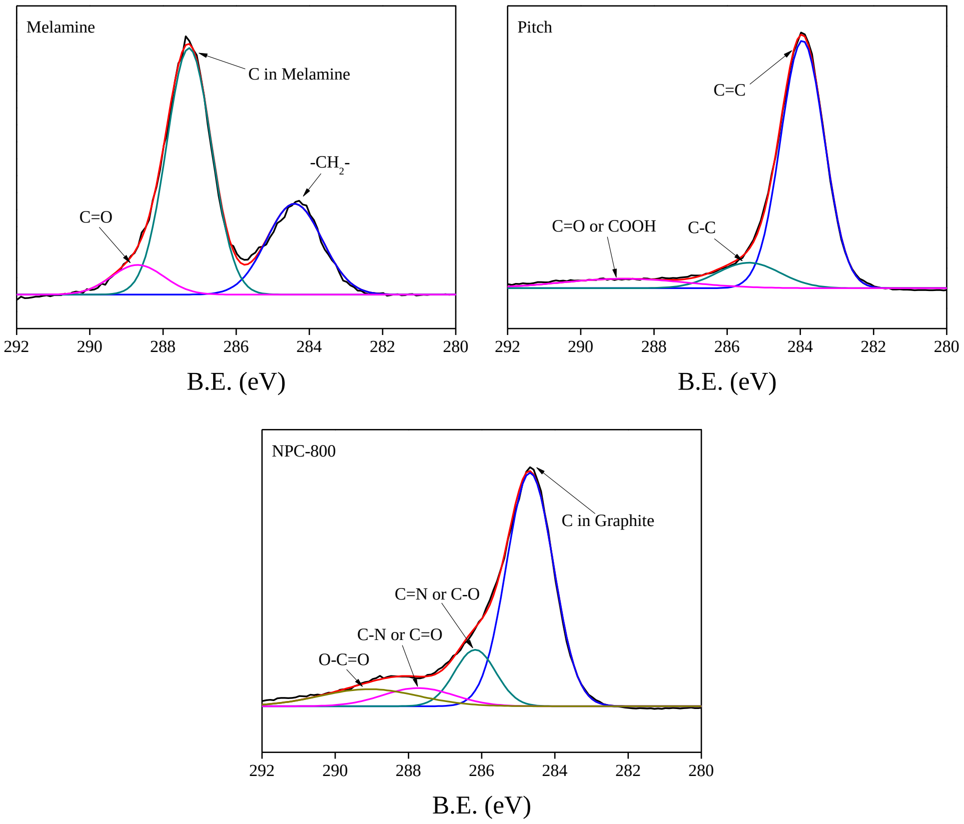

Figure S2. C1s XPS spectra of precursors (pristine melamine and pitch) and NPC-800.

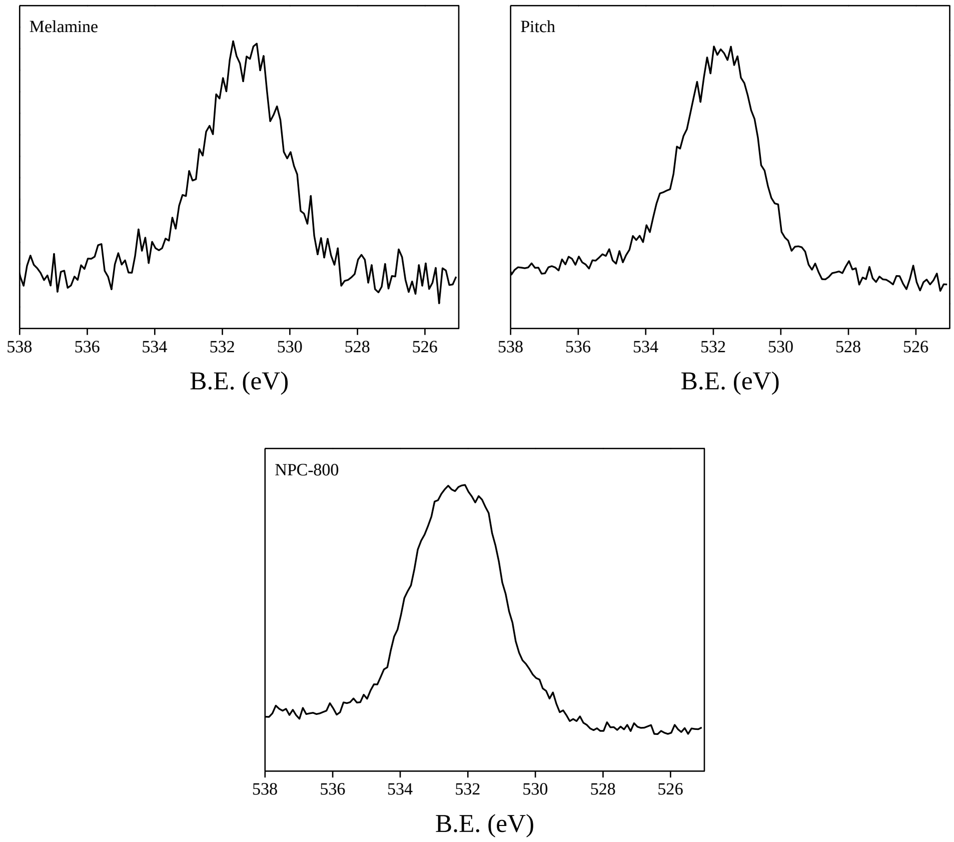

Figure S3. O1s XPS spectra of precursors (pristine melamine and pitch) and NPC-800.

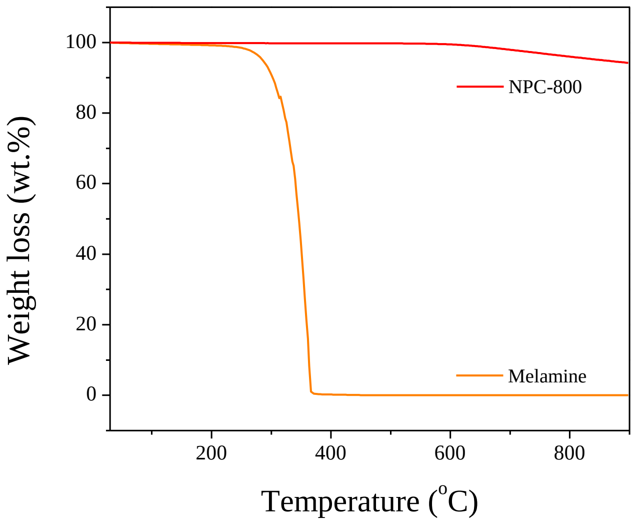

Figure S4. TGA curves for pristine melamine and NPC-800 measured at increasing temperature under N_2 flow.

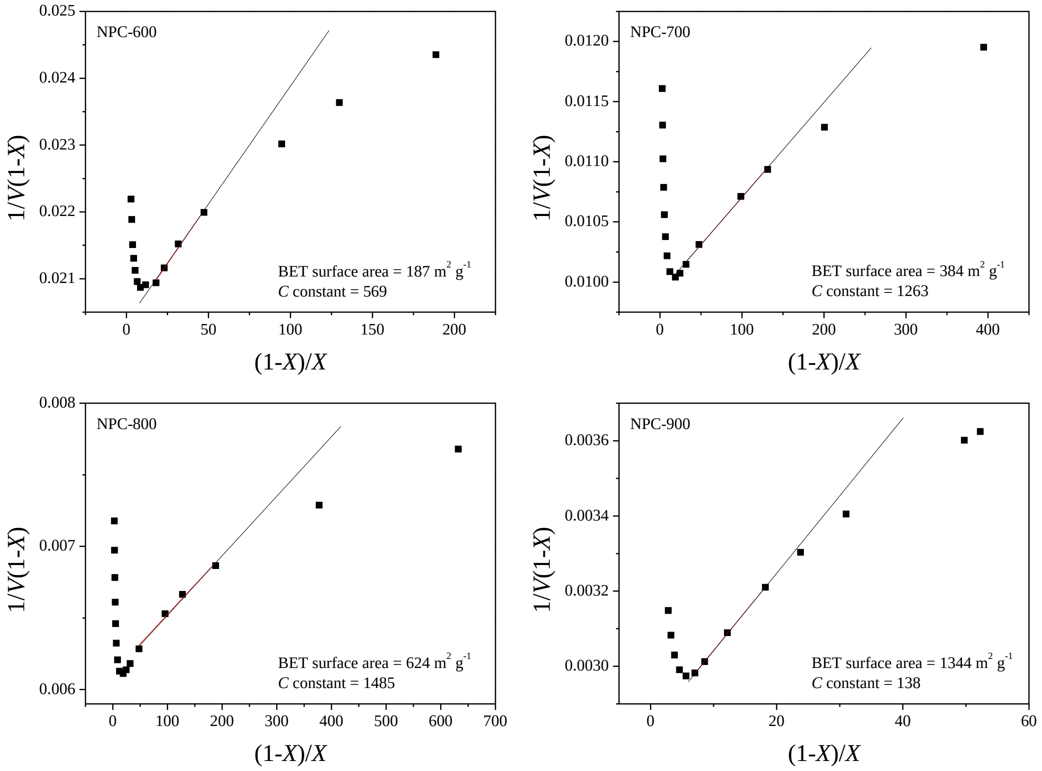

Figure S5. BET plots for series of NPCs, using alternative equation, from Ar isotherms at 87 K.

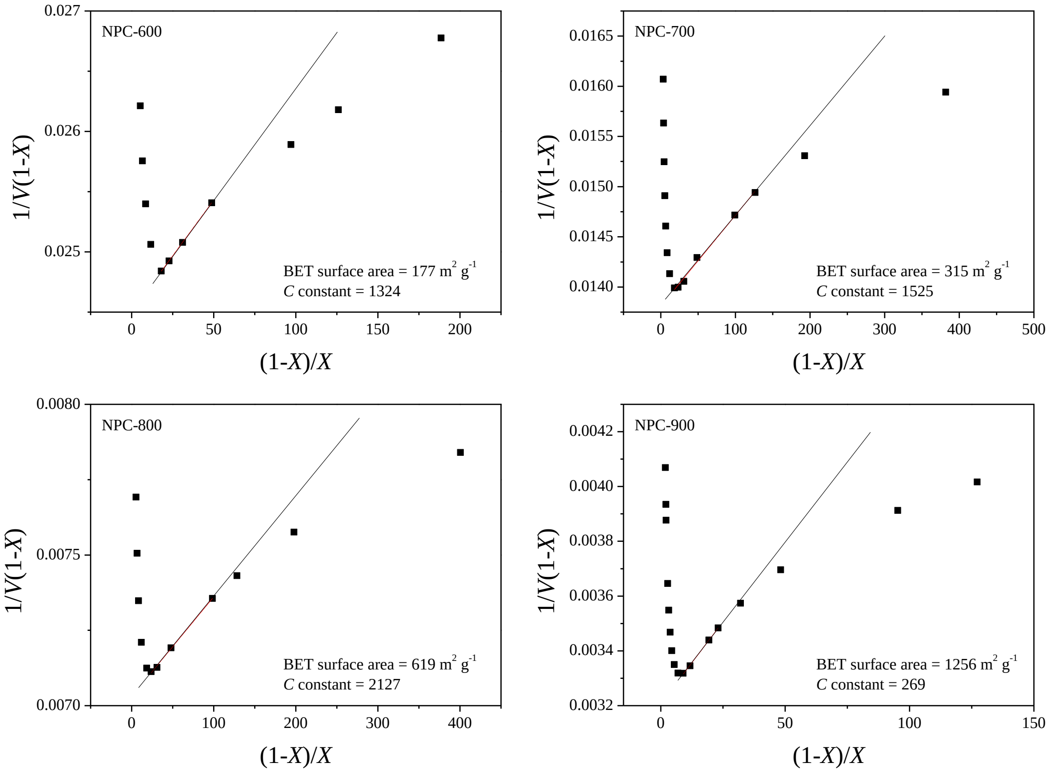

Figure S6. BET plots for series of NPCs, using alternative equation, from N_2 isotherms at 77 K.

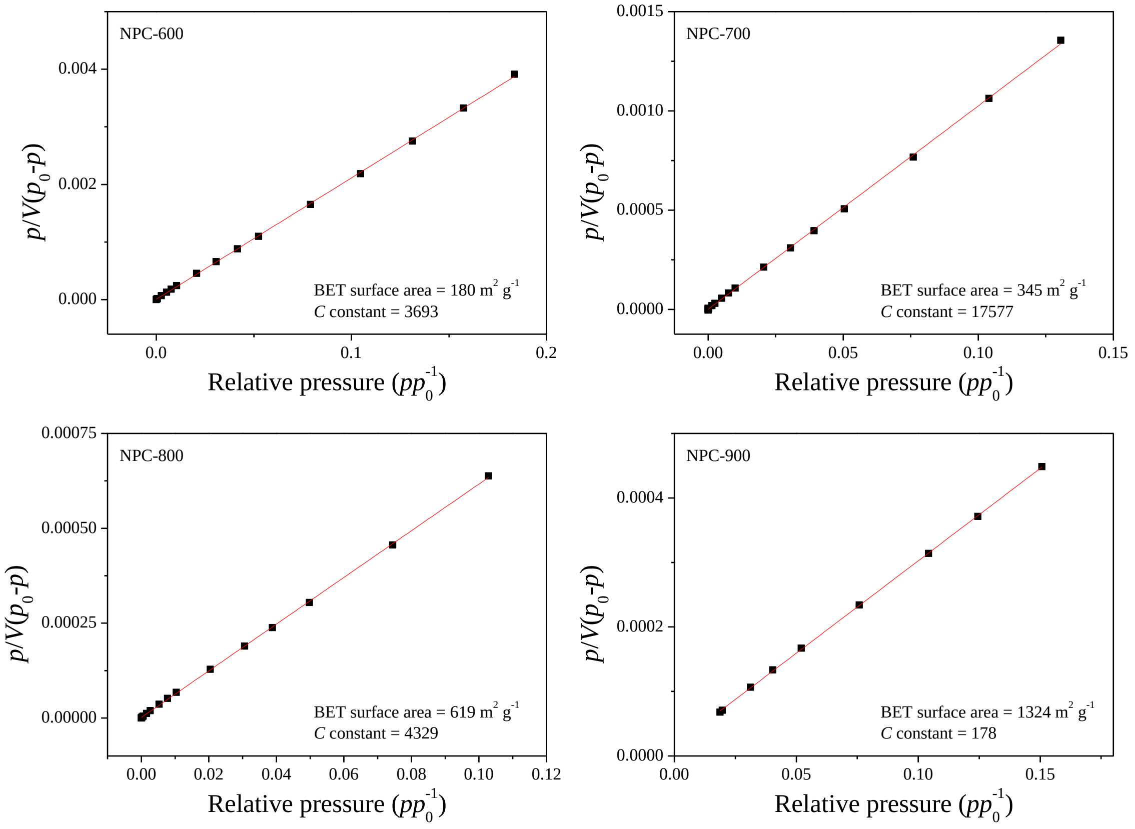

Figure S7. BET plots for series of NPCs, using standard equation, from Ar isotherms at 87 K.

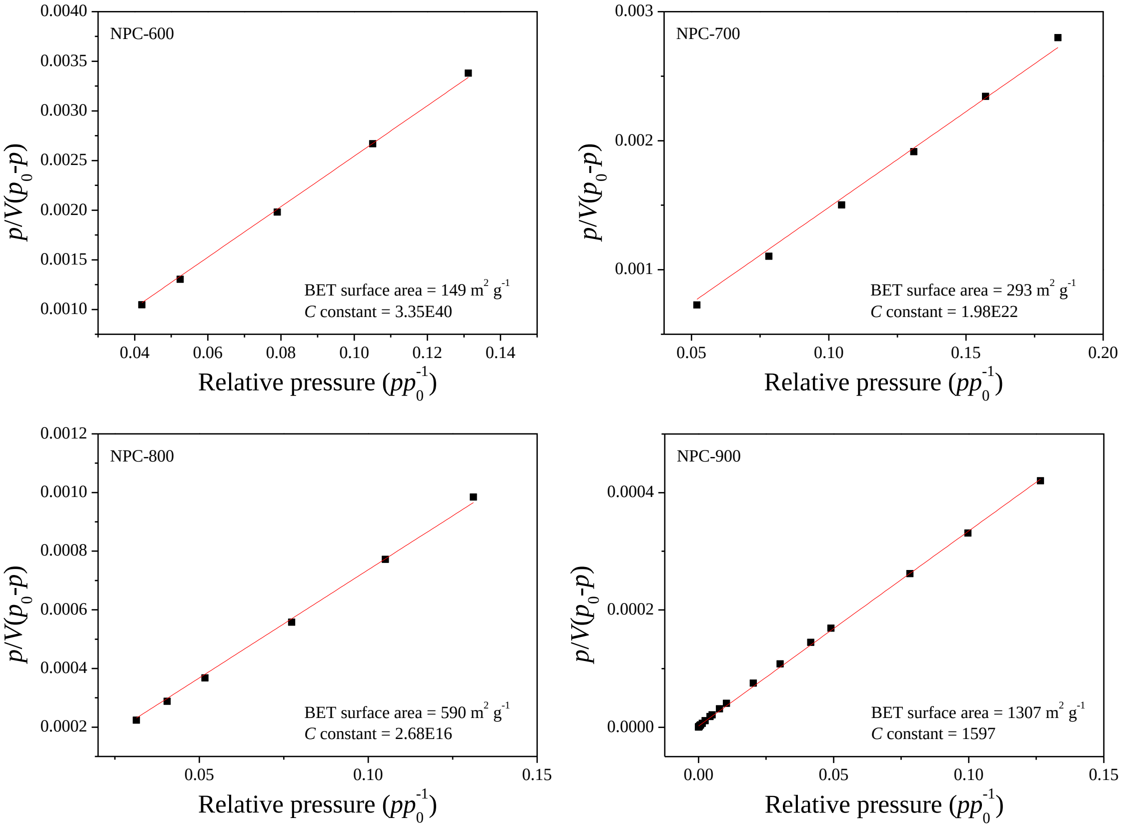

Figure S8. BET plots for series of NPCs, using standard equation, from N_2 isotherms at 77 K.

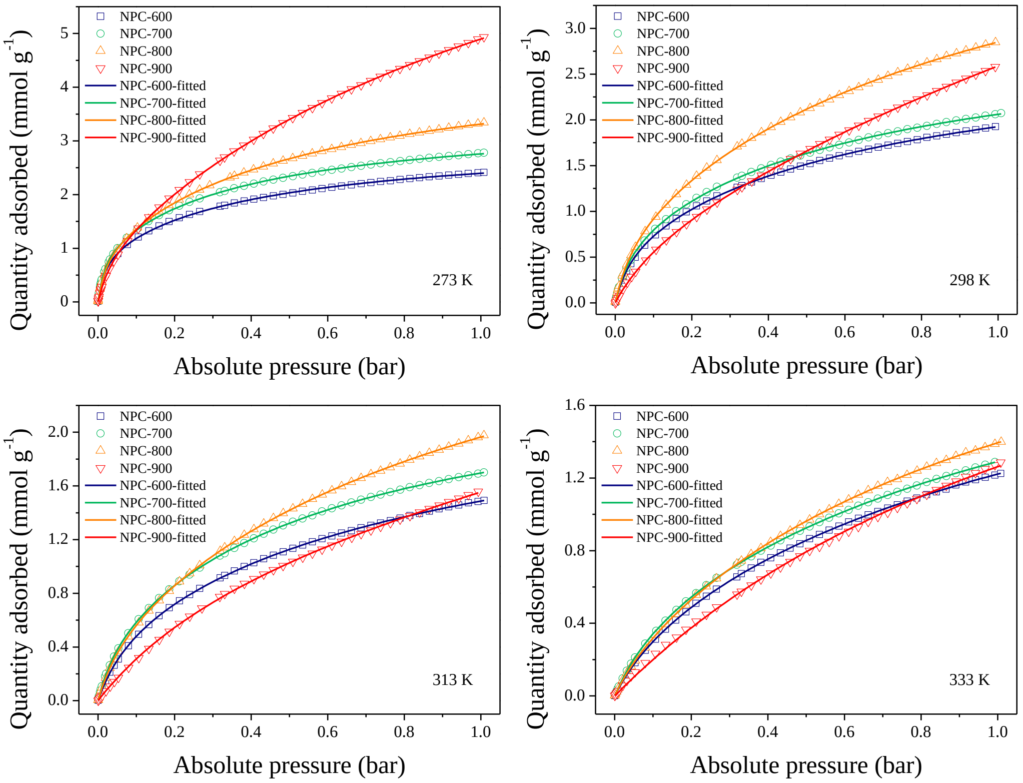

Figure S9. CO_2 adsorption isotherms for NPCs at 273, 298, 313, and 333 K. Plots are experimental data and lines show DSL model correlations.

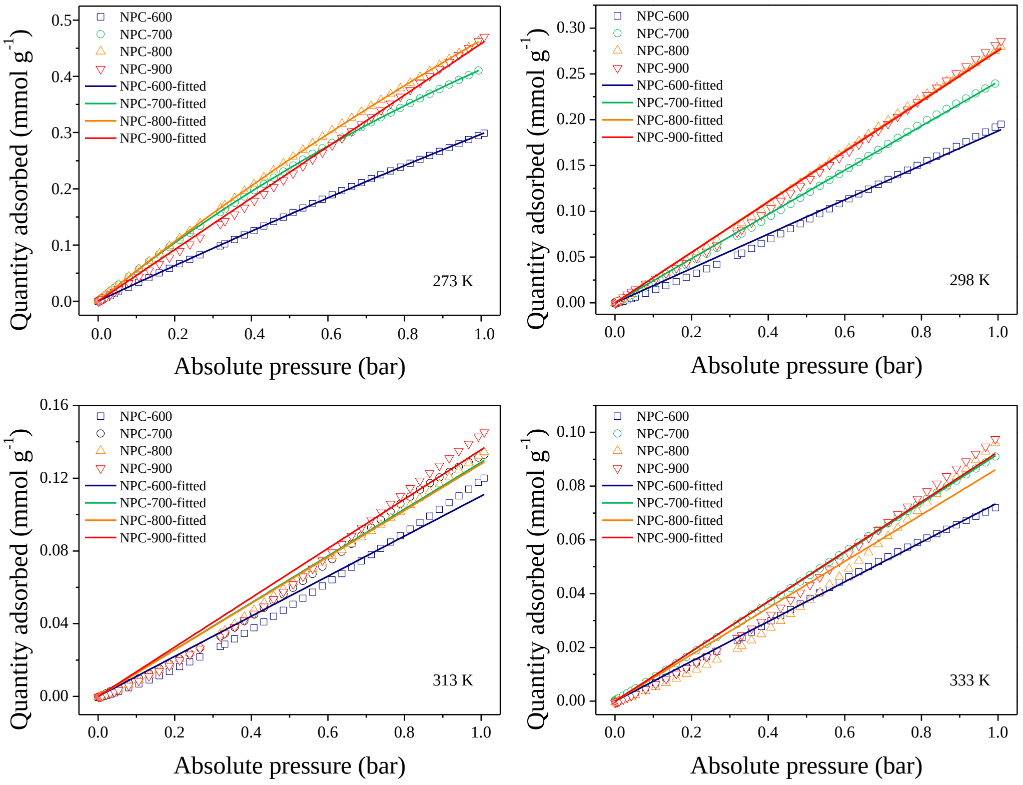

Figure S10. N_2 adsorption isotherms for NPCs at 273, 298, 313, and 333 K. Plots are experimental data and lines show SSL model correlations.

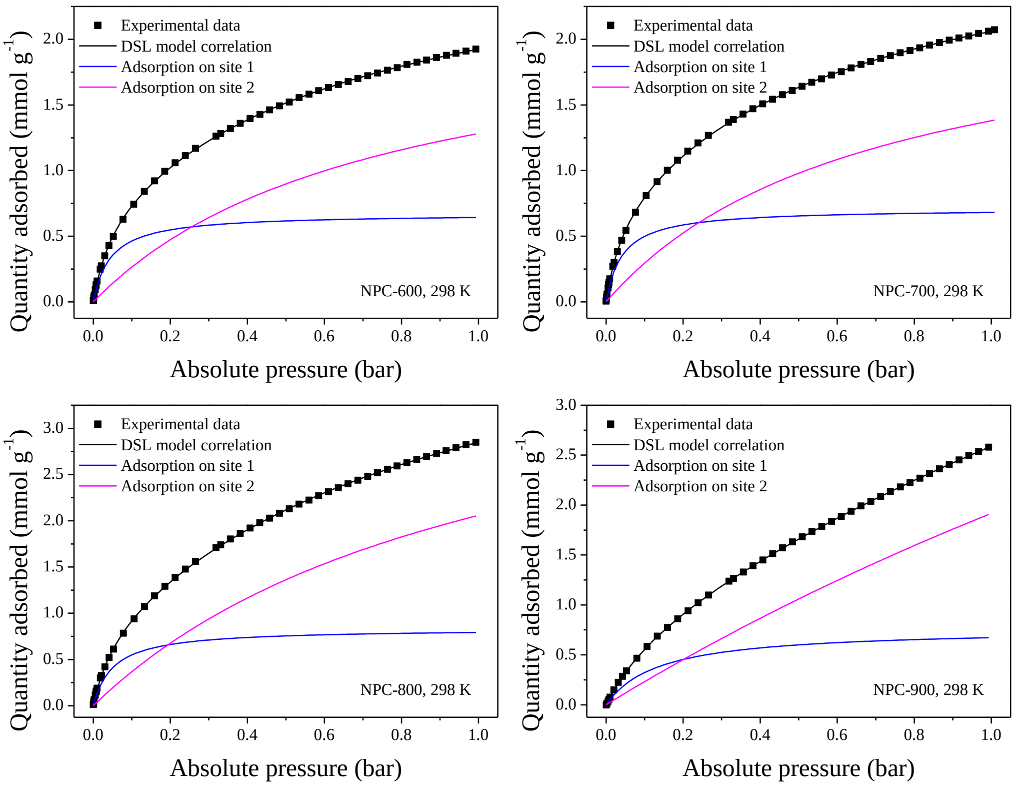

Figure S11. CO_2 adsorption isotherms for NPCs at 298 K. Plots are experimental results. Black, blue, and magenta lines show DSL model correlation, adsorption at site 1, and adsorption at site 2, respectively.

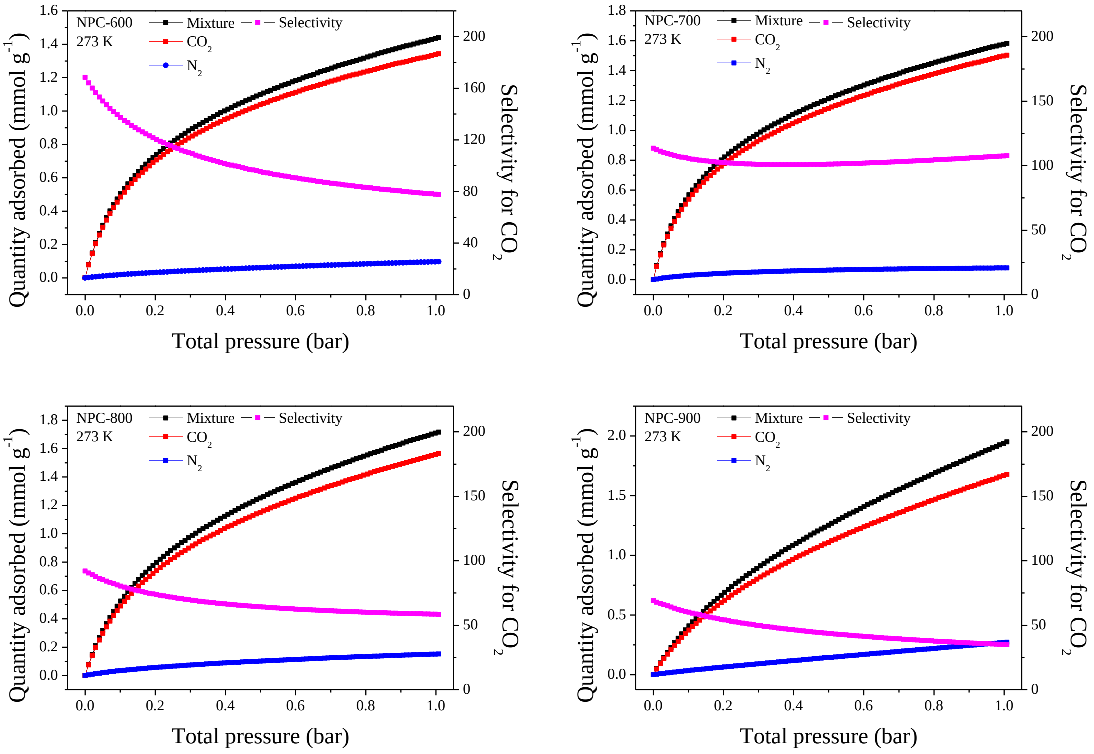

Figure S12. Binary IAST isotherms and mixture adsorption selectivities for CO_2 over N_2 at 273 K on NPCs.

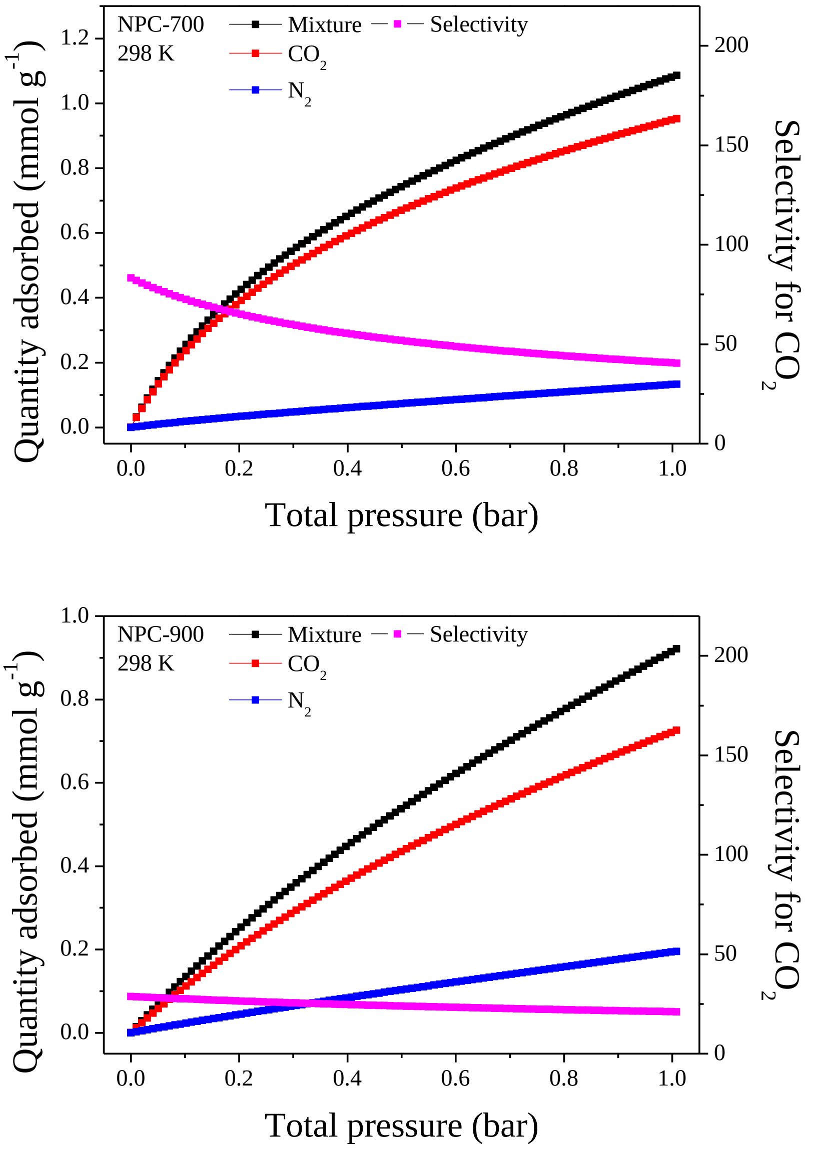

Figure S13. Binary IAST isotherms and mixture adsorption selectivities for CO_2 over N_2 at 298 K on NPC-700 and NPC-900.

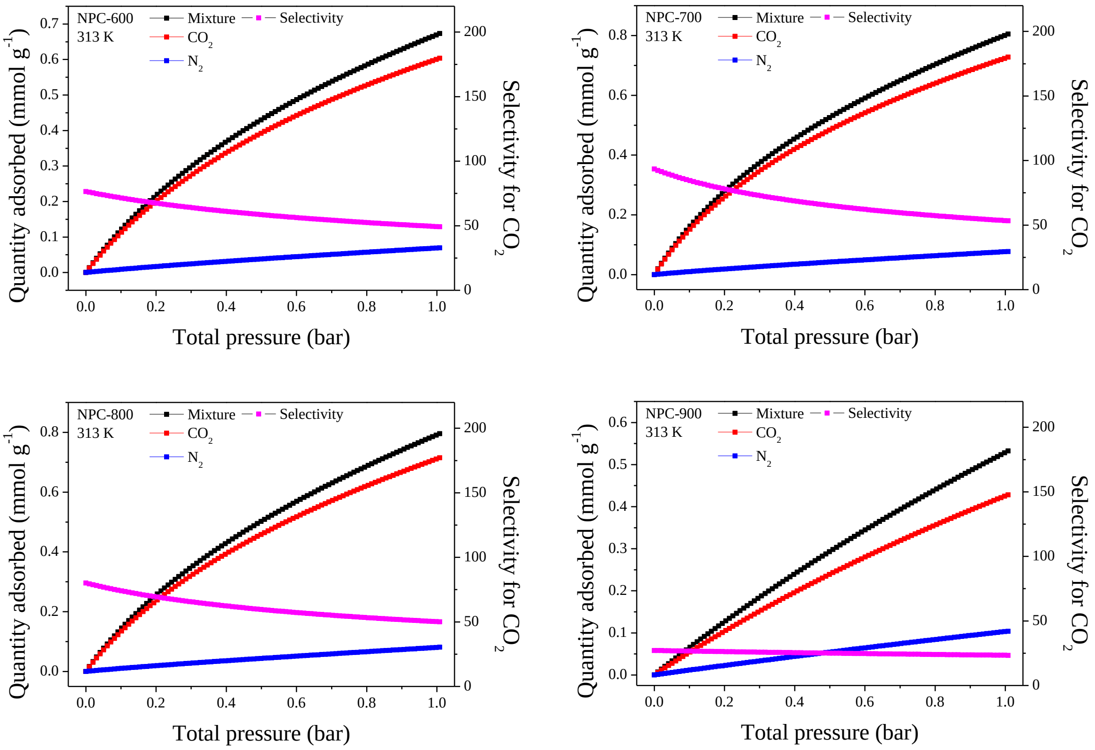

Figure S14. Binary IAST isotherms and mixture adsorption selectivities for CO_2 over N_2 at 313 K on NPCs.

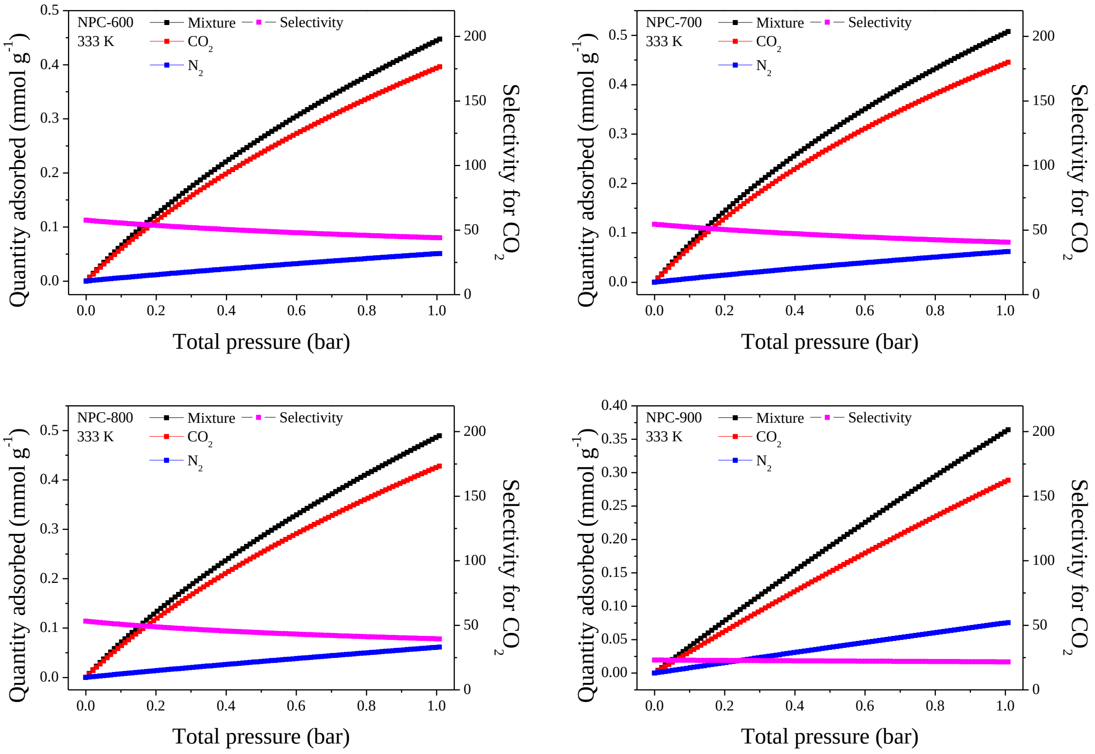

Figure S15. Binary IAST isotherms and mixture adsorption selectivities for CO_2 over N_2 at 333 K on NPCs.

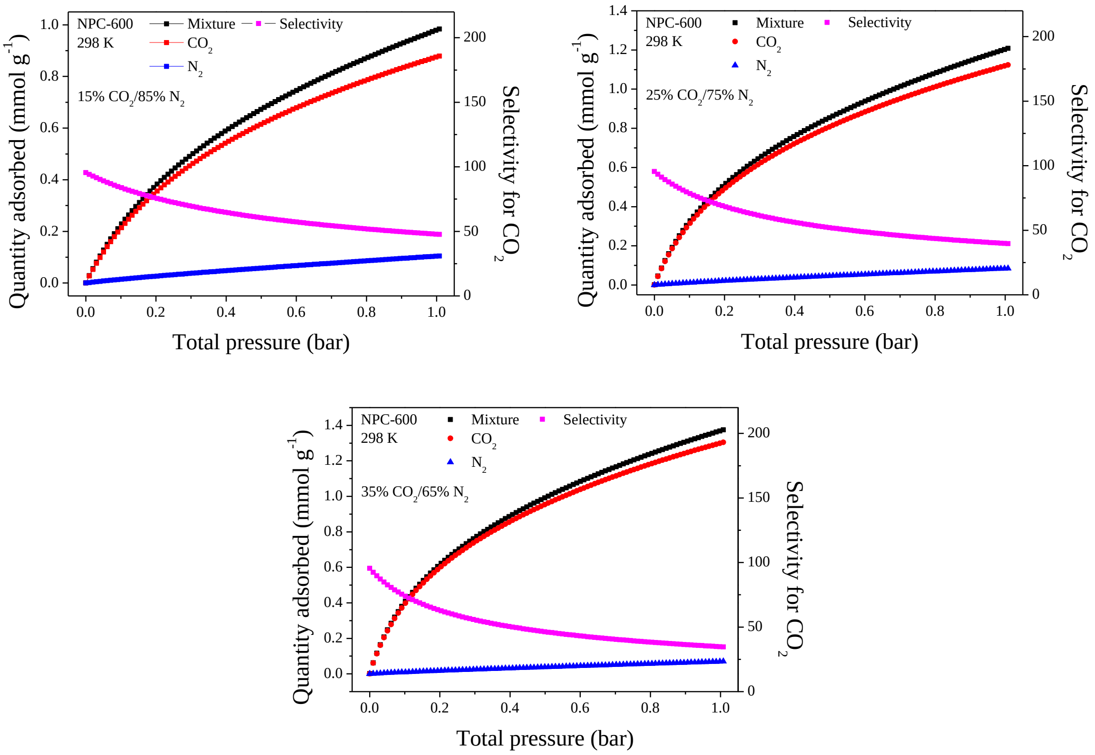

Figure S16. Binary IAST isotherms and CO_2/N_2 selectivities, according to CO_2 ratio, over N_2 at 298 K on NPC-600.

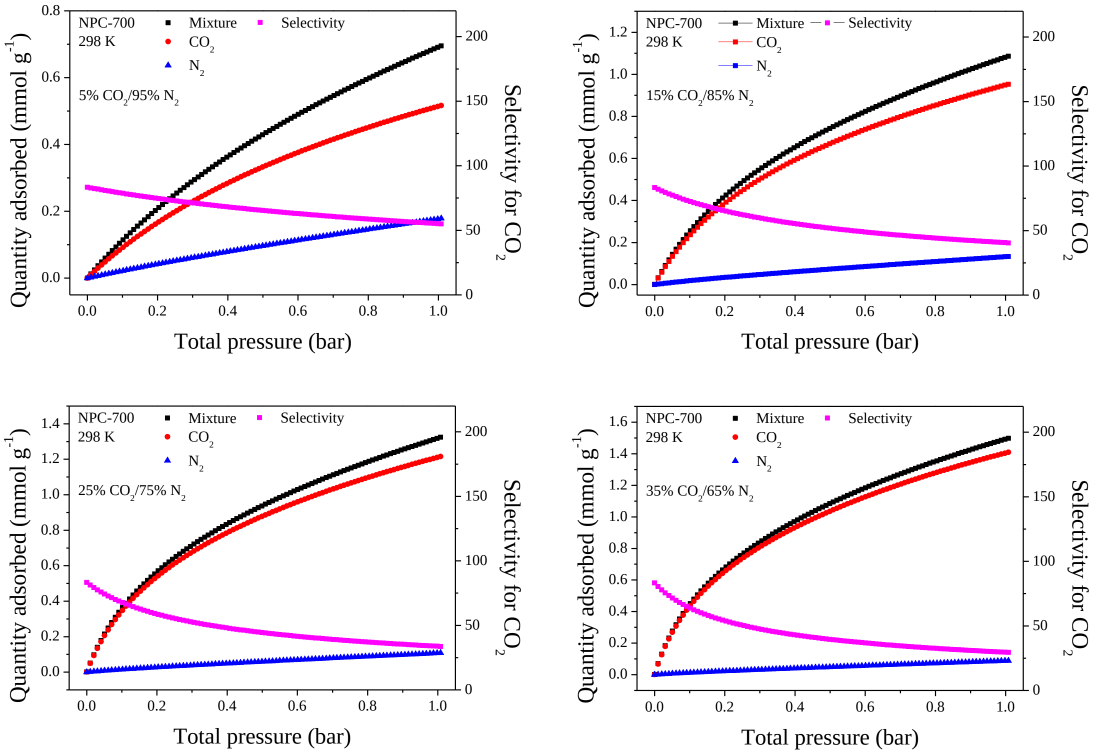

Figure S17. Binary IAST isotherms and CO_2/N_2 selectivities, according to CO_2 ratio, over N_2 at 298 K on NPC-700.

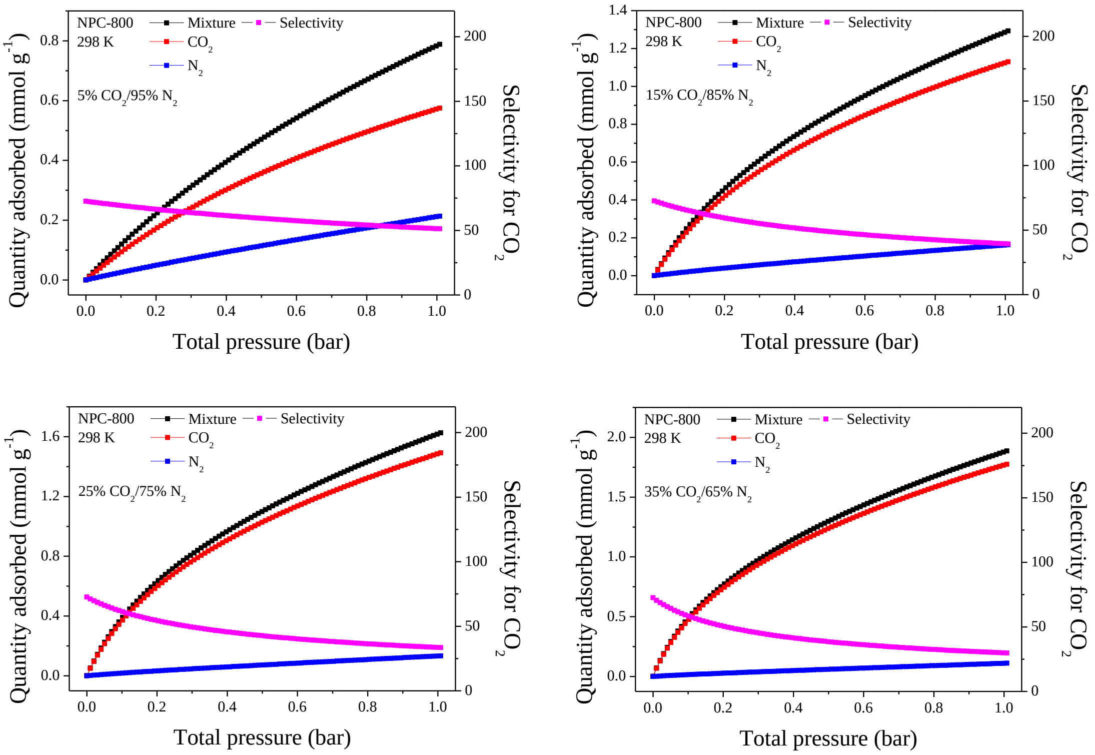

Figure S18. Binary IAST isotherms and CO_2/N_2 selectivities, according to CO_2 ratio, over N_2 at 298 K on NPC-800.

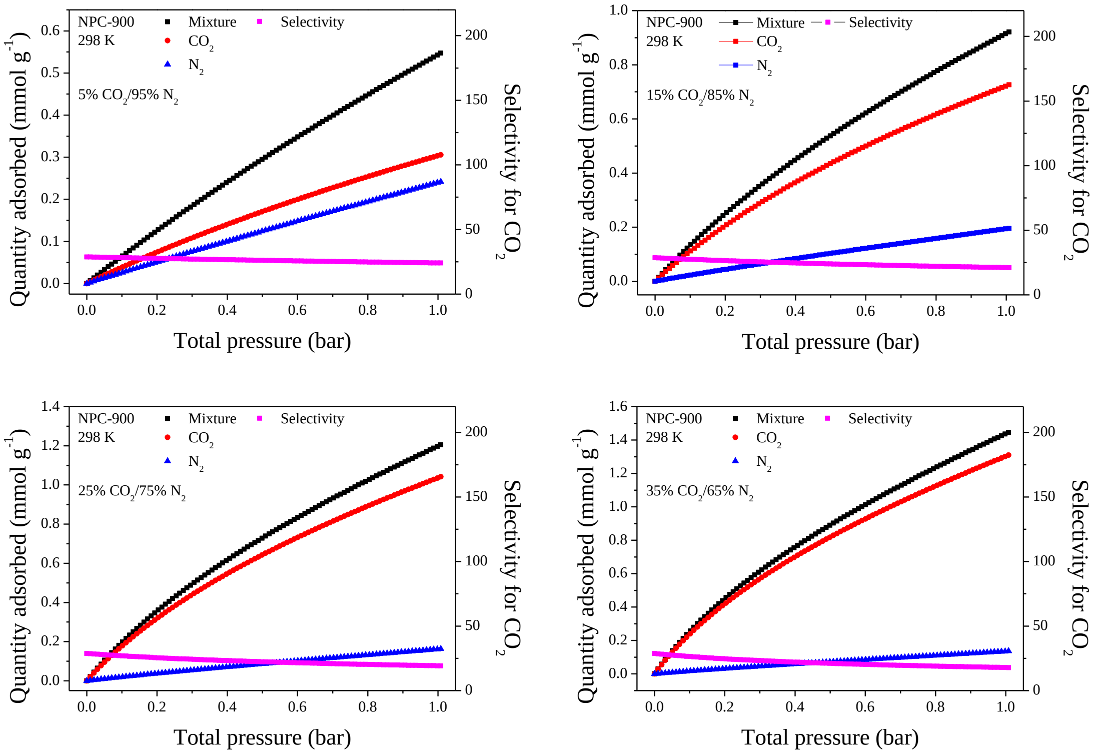

Figure S19. Binary IAST isotherms and CO_2/N_2 selectivities, according to CO_2 ratio, over N_2 at 298 K on NPC-900.

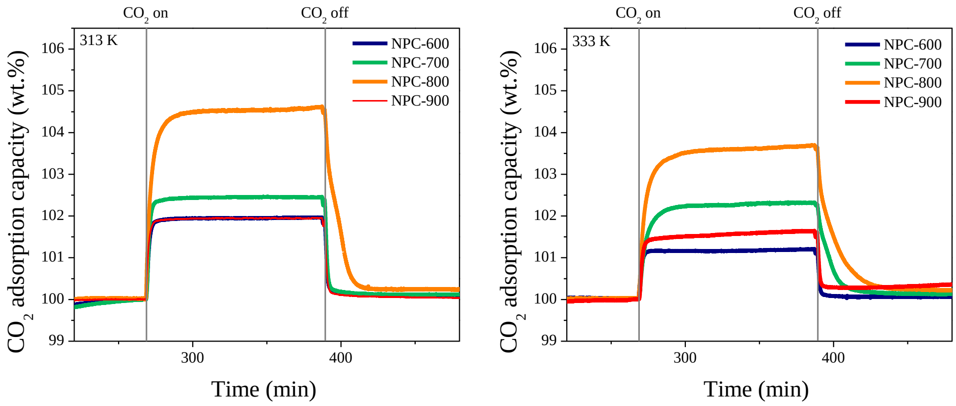

Figure S20. Time-dependent CO_2 adsorption curves of NPCs at 313 and 333 K.
